# Life satisfaction, cardiovascular risk factors, unhealthy behaviours and socioeconomic inequality, 5 years after coronary angiography

**DOI:** 10.1186/s12889-015-2047-0

**Published:** 2015-07-15

**Authors:** Michèle Baumann, Anastase Tchicaya, Kyle Vanderpool, Nathalie Lorentz, Etienne Le Bihan

**Affiliations:** Research Unit INSIDE, Institute Health & Behaviour, University of Luxembourg, Luxembourg City, Luxembourg; Luxembourg Institute of Socio-Economic Research (LISER), Luxembourg City, Luxembourg

**Keywords:** Cardiovascular disease, Life satisfaction, Behavioural risk factors, Social inequalities, Intention to change

## Abstract

**Background:**

Five years after coronary angiography, life satisfaction (LS) among patients may be related to incidents of cardiovascular diseases, risk factors and unhealthy behaviours and socioeconomic conditions, but their respective influence remains unclear. Our aim is to analyze LS and its relationships with those factors.

**Methods:**

Among the 4,391 patients initially contacted, 547 deaths were reported and 209 had an invalid address. In 2013–2014, 3,635 patients who underwent coronary angiography in 2008–2009 at the *National Institute of Cardiac Surgery and Cardiological Intervention* (INCCI) in Luxembourg were asked to complete a self-administered questionnaire assessing LS [1–10] and other variables. Data were analysed via multiple regression models adjusted initially on age, sex and income, and for a second time with the addition of all CVRF.

**Results:**

LS of 1,289 volunteers (69.2 years) was 7.3/10. Most were men, Luxembourgish, employees and manual workers, had secondary education and an income of 36,000 euros or more per year. LS was lowest in female patients, and those with a low to middle income. Patients who lived in a couple had the best LS. Patients with a history in the previous 5 years of physical inactivity (regression coefficient: −0.903), angina pectoris (rc −0.843), obesity (rc −0.512), diabetes, or hypercholesterolemia, were more likely to have lower LS. The previous associations were mostly maintained on the second analysis, with the exceptions of diabetes and obesity. In addition, patients who stopped smoking because of peer pressure (rc −0.011) had a lower LS.

**Conclusions:**

The finding that LS was lowest among female patients calls for further research on symptoms, and potential risk factors. Also, certain patient profiles are linked with low LS: ‘inclined abstainers’ who intended to modify their behaviours, but could not do so, and ‘disinclined abstainers’ who had no intention of changing and were insufficiently concerned to do so. Patients who stopped smoking and perceived it as unpleasant also had low LS. ‘Disinclined actors’ were those patients who had to adjust their lifestyles, but were ambivalent about their intentions and the behaviour, which they continued. Health promotion programs would benefit from targeting factors that moderate the unfavourable intention-behaviour relationship and can help enhance LS.

## Background

Cardiovascular diseases (CVD) are a high priority for European health and social policy makers due to their high prevalence and the fact that they cause long-term disabilities [1]. Cardiovascular risk factors and unhealthy behaviours (CVRF) such as genetic factors, lifestyle, smoking, alcohol consumption and cultural or economic circumstances may cause different impairment/deficiency profiles. It is well established that early management of CVRF by lifestyle modification and/or therapeutic intervention leads to a marked reduction in mortality and morbidity [2].

A recent study of coronary heart disease [3] examined the relationship with life satisfaction (LS) and found that LS was associated with angina, but not myocardial infarction. In this research, LS was defined as the average of seven satisfaction domains: job, family, sex life, self-satisfaction, love relationship, leisure activities, and standard of living, but only four were related to angina: job, family, sexual, and self-satisfaction. In another study with other psychological constructs, stress was related to angina, but not ‘objective’ cardiovascular-related outcomes [4]. This previous work assessed particular life domains (i.e. evaluated specific areas of life), while other studies used a global measure (i.e. evaluation of life in general), which is an overall perceptual measure of the degree of discrepancy between individual aspirations and achievements or contentment [5, 6]. The committee of the third Eurofound survey considers monitoring of LS to be a key element of the social progress of Europeans [7]. Long-term cardiovascular disabilities are not exclusively physical, but also involve psychological distress, social and familial repercussions on daily life, restrictions in routine, leisure and work activities, and substantial socioeconomic impact. All these consequences constitute a challenge for individuals trying to maintain autonomy and an acceptable level of LS. We therefore opted here for a general approach to LS in the belief that risk factors, unhealthy behaviours, and socioeconomic conditions are all related to the LS of CVD patients.

In the chronic phase, cardiovascular risk management, lifestyle changes, secondary prevention of relapses among known patients and treatment of heart attacks are well documented to lead to decreases in the originally very high case fatality of myocardial infarctions [8]. When health-related interventions are applied to reduce hypertension, smoking, diabetes, poor diet, physical inactivity and obesity [9], we can suppose that the LS of patients may be affected. After 5 years, study patients living at home with their families may well have reorganised their daily lives, adapted to their new preventive behaviours, adjusted their lifestyles, and rearranged their plans for the future - all in the context of increased health inequalities between groups with different socioeconomic profiles. The question of how the socioeconomic conditions in which people live lead to inequalities in health has been well documented in the general population [10, 11], but less so in the population with CVD [12]. Socioeconomic factors are known to potentially impact on health-related behaviours (smoking, unhealthy diet), but the consequences for LS remain under-researched. Improvements in our comprehension of the cognitive aspects of CVD make it clear that what happens in the mind has to be taken into account if we want to understand how the socioeconomic circumstances in which people live influence health [12].

A study among all patients who underwent coronary angiography in 2008 at the *Institut National de Chirurgie Cardiaque et de Cardiologie Interventionnelle* (INCCI) in Luxembourg [13] observed lack of awareness of risk factors. Only 8 % of men and 7 % of women could cite at least three risk factors. This knowledge ranged from 4 % of patients with primary level education to 11 % and 20 % of those with secondary and university education, respectively. More than 1 in 10 patients could name no CVRF. However, researchers [14, 15] attempted to account for the gap between knowledge of risk factors and change in unhealthy behaviours. Crossing the level of intention and subsequent practice, their theoretical approach identified four profiles [14, 16]: ‘inclined actors’ who are motivated to change their behaviour and act to do so; ‘inclined abstainers’ who are willing to change, but refrain from acting; ’disinclined actors’ who are unwilling, but act anyway; and ‘disinclined abstainers’ who are unwilling and refrain from acting. The findings indicate that intention is a moderate predictor of behaviour and the gap between intention and behaviour is caused by high intenders not taking action [15].

In the Grand-Duchy of Luxembourg, CVD was the primary cause of mortality and a major reason for hospital admission in 2012. This has led public health authorities to consider CVD as one of their national healthcare priorities [17]. In terms of the number of years of life lost due to premature death, cardiovascular disease was the highest-ranking cause in 2010, and the principal cause of acquired long-term disabilities [2]. Five years after undergoing coronary angiography, critical questions may be raised about what incidences of cardiovascular diseases, risk factors, unhealthy behaviours, and socioeconomic conditions are related to LS. Knowledge of these issues may be useful for the social and public health systems developing educational interventions for patients and families. The survey further analysed, 5 years post-event, the LS of patients living at home in Luxembourg, and its respective associations with the previously mentioned factors.

## Methods

### Study design, sample and recruitment

This survey was a retrospective health record audit of the INCCI, involving all cardiovascular patients who underwent coronary angiography between 2008 and 2009. Among the 4,391 patients initially contacted, 547 deaths were reported and 209 had an invalid address. In 2013–2014, 3,635 patients were sent a letter that contained information about the aims of the survey and invited them to complete a self-administered questionnaire in the language of their choice: Portuguese, French or German.

### Description of the diagnostic characteristics of the patients in 2008

The diagnoses were mostly angina pectoris 43.2 %, chronic ischemic cardiomyopathy 14.3 %, acute infarction of the myocardium 8.8 %, anomalies of breathing 6.5 %, endocarditis 5.1 %, and myocardiopathy 4.5 % [13].

### Instruments and their translation

As Luxembourg is multilingual and very culturally diverse (more than 170 different nationalities), our questionnaires were available in the three languages of the country. Each version was translated and back-translated, and proofread by native-speaking professional translators. All cardiologists from the Luxembourgish Institute speak many languages, are culturally diverse, and were trained elsewhere in Europe. They collaborated in supervising the conception of all documents, and their translation.

### Ethical approval

The protocol was approved by the National Committee of Research Ethics and the Committee for Data Protection of Luxembourg.

### Data collected

#### Life satisfaction (LS) (dependent variable)

As in the European Foundation survey 2009–2010 [6], LS assessment was based on one question, which was easy to routinely use. Each respondent self-rated the degree of their level of satisfaction with life on a scale from 1 to 10; 10 being the highest.

### Incidences of cardiovascular diseases

In the previous 5 years, CVD data were gathered for bypass surgery, myocardial infarction and/or angina pectoris.

### Cardiovascular risk factors

Diabetes, hypertension, hypercholesterolemia, weight and height were self-reported. Based on the International Obesity Task Force [18], convened by the World Health Organization, subjects were classified on the basis of Body Mass Index (BMI) as obese (≥30.0 kg/m2), overweight (25.0–29.9 kg/m^2^) or normal (<25.0 kg/m^2^).

### Unhealthy behaviours

Patients were asked using separate questions if they had changed their behaviour concerning tobacco consumption, physical activity or eating habits over the previous 5 years. When their behaviour had improved with regard to CVRF, i.e. they had stopped smoking, increased their physical activity or paid more attention to their eating habits, we asked, separately for each change, for the reasons for modifying the behaviour. The three reasons proposed were: ‘Because of the cardiovascular disease’, ‘Fear of the consequences for health’, and ‘Under the influence of, or at the request of, others’.

### Socioeconomic characteristics

Information was collected on age, sex, living in a couple, nationality, educational level (primary, secondary, and tertiary), professional status (manual worker, employee, executive, other), retirement status, and total annual income in euros (low, low to middle, high to middle, and high).

### Statistical analysis

We performed multiple regression models to determine the separate effects of each group of variables on LS. First, the model was adjusted with age, sex and income. The other socio-demographic variables (nationality, marital status, and level of education, professional status and occupation) were introduced one by one, as were the incident cardiovascular diseases, the cardiovascular risk factors and the reasons for change in behaviour. We then followed the same approach by adding risk factors (diabetes, hypertension, hypercholesterolemia, tobacco consumption, physical activity and eating habits) to previous adjustment factors. All analyses were conducted with the statistical software SAS (PROC GLM).

## Results

Among the 4,391 patients initially contacted, 547 deaths were reported and 209 had an invalid address. We received 1,289 completed questionnaires, leading to a rough response rate estimate of 35.5 % (1,289/3,635).

The LS of the participants was 7.3/10 with a mean age of 69.2 years. The majority of the patients were men, Luxembourgish, employees and manual workers, had been educated to a secondary level and lived on an annual income of 36,000 euros or more (left-hand column of Table 1). Adjusted on age, sex and income, LS was lowest in female patients, those not living in a couple or with a low to middle income (centre column of Table 1). Adjusted on age, sex, income and all risk factors, most previous relationships (right-hand column of Table 1), were maintained, with the exception of not living in a couple.

**Table 1:** Description of socioeconomic characteristics (left-hand column) of patients and regression model on life satisfaction adjusted with age, sex and income (centre column) and adjusted on age, sex, income and all CVRF (right-hand column)

		Simple description mean (s.d.) or %	Life satisfaction [1–10]^1^ Estimate p^3^		Life satisfaction [1–10]^2^ Estimate p^3^	
Life satisfaction	[1–10]	7.3 (2.1)				
Age		69.2 (11.1)	0.010	0.063	0.009	0.173
Sex	Women	28.7	−0.458	0.001**	−0.406	0.016*
	Men	71.3	0.000		0.000	
Nationality	Luxembourgish	77.5	0.126	0.404	0.028	0.867
Marital status	Living in couple	74.0	0.352	0.021*	0.326	0.071
Level of education	Primary	31.9	−0.167	0.478	−0.314	0.213
	Secondary	52.1	−0.223		−0.358	
	Higher	16.0	0.000		0.000	
Professional status	Manual worker	24.5	0.055	0.857	0.039	0.987
	Employee	37.5	0.136		0.052	
	Executive	24.0	−0.001		0.092	
	Other	13.9	0.000		0.000	
Occupation	Retired	78.1	0.205	0.297	0.239	0.293
Annual Income (euros)	<18 000	4.7	−1.055	0.000***	−0.705	0.030*
	18 000–35 999	29.2	−0.826		−0.502	
	36 000–53 999	33.7	−0.282		−0.214	
	54 000 +	32.4	0.000		0.000	

^1^Regression model adjusted with age, sex and income. Other variables were introduced one by one

^2^Regression model adjusted with age, sex, income and risk factors. Other variables were introduced one by one

^3^Type III F Tests. Significant p-value: *p < 0.05; **p < 0.01; ***p < 0.001

Most had a history of bypass surgery, myocardial infarction, hypercholesterolemia, hypertension and obesity/overweight. Ten percent were former smokers; 49 % had stopped smoking in the previous 5 years, for fear of consequences for health. Sixty–six percent declared a physical activity and 71 % ‘paid more attention to their eating habits’ (left-hand column of Table 2). Adjusted on age, sex and income, the patients who were not physically active (regression coefficient (rc) –0.903), and/or had angina pectoris (rc −0.843), obesity (rc −0.512), diabetes, and hypercholesterolemia were more likely to have lower LS (centre column of Table 2). Adjusted on age, sex, income and all risk factors, most previous associations (right-hand column of Table 2), were maintained, with the exceptions of diabetes and obesity. Additionally, patients who stopped smoking because of peer pressure (rc −0.011) had lower LS.

**Table 2:** Description of incident CV, risk factors and unhealthy behaviours and reasons for changes in behaviour (left-hand column) of patients and regression model on life satisfaction adjusted with age, sex and income (centre column) and adjusted on age, sex, income and all CVRF (right-hand column)

		Simple description mean (s.d.) or %	Life satisfaction [1–10]^1^ Estimate p^3^		Life satisfaction [1–10]^2^ Estimate p^3^	
*Incidents of cardiovascular diseases in the previous 5 years:*						
- Bypass surgery	Yes	21.2	−0.013	0.935	−0.002	0.990
- Myocardial infarction	Yes	12.0	−0.053	0.804	0.012	0.961
- Angina pectoris	Yes	10.6	−0.843	0.000***	−0.763	0.002**
*CV risk factors:*	Yes	29.0	−0.336	0.023*	−0.268	0.114
- Diabetes						
- Hypertension	Yes	43.1	−0.257	0.056	−0.118	0.473
- Hypercholesterolemia	Yes	48.4	−0.307	0.018*	−0.300	0.049*
- Body mass index (BMI)	Obesity	31.8	−0.512	0.001**	−0.327	0.152
	Overweight	44.1	0.005		−0.038	
	Normal	24.1	0.000		0.000	
*Unhealthy behaviours:*	Smoker	10.0	−0.096	0.648	−0.111	0.645
- Tobacco consumption						
	Non-smoker	90.0	0.000		0.000	
*Reason for stopping smoking*:						
Cardiovascular diseases	Yes	31.6	−0.377	0.055	−0.258	0.252
Fear of consequences on health	Yes	48.7	−0.056	0.755	−0.017	0.934
Peer pressure	Yes	15.7	0.229	0.344	0.534	0.046*
- Physical activity	No	33.9	−0.903	0.000***	−0.678	0.000***
	Occasional	23.6	−0.306		−0.311	
	Regular	42.5	0.000		0.000	
*Reason for increasing physical activity:*						
Cardiovascular diseases	Yes	36.0	0.341	0.172	0.373	0.213
Fear of consequences on health	Yes	43.4	−0.147	0.556	−0.236	0.426
Peer pressure	Yes	6.4	−0.089	0.859	−0.011	0.984
- Pay attention to eating habits	Yes	71.0	−0.060	0.633	0.089	0.529
*Reason for changing eating habits:*						
Cardiovascular diseases	Yes	46.3	0.030	0.866	0.152	0.458
Fear of consequences on health	Yes	42.7	−0.060	0.737	−0.012	0.952
Peer pressure	Yes	8.3	0.063	0.843	0.387	0.237

^1^Regression model adjusted with age, sex and income. Other variables were introduced one by one

^2^Regression model adjusted with age, sex, income and risk factors. Other variables were introduced one by one

^3^Type III F Tests. Significant p-value: *p < 0.05; **p < 0.01; ***p < 0.001

## Discussion

Five years after a coronary angiography, this research examined the respective associations between incidents of CVD, risk factors, unhealthy behaviours, and socioeconomic inequality and life satisfaction (LS) among INCCI patients in Luxembourg.

Patients suffering from angina pectoris in the previous 5 years had lower LS than patients with myocardial infarction and/or bypass surgery. A previous study found the same results; LS related to angina was probably principally responsible for this association [3]. Previous work suggests that angina is a predictor of future cardiovascular events, but other investigations of psychological factors and coronary heart disease find that conventional risk factors explain little of the observed association [19]. Of course, having a chronic illness or a disability is associated with reduced LS, and the effect is larger if this disability limits daily activities. Those findings accord with the conclusion of the last report of Eurofound, which observed that the most important predictor of LS is health [7].

Another finding consistent with recent research [3] was the relationship between CVRF and LS. Patients who were not physically active and suffered from obesity, diabetes and hypercholesterolemia were more likely to have low LS. Among the initial population of our study, the investigation observed that awareness of hypercholesterolemia was more often declared as a CVRF than diabetes [13]. What can we conclude in regard to the theoretical perspective cited in the introduction. Two profiles of patients were linked with lowest LS: those who were ‘inclined abstainers’ and had an intention to modify their behaviours, but could not do it, and those who were ‘disinclined abstainers’ and had no intention to change because they were not concerned about continuing the behaviour. A recent research study among Luxembourgish people showed that more time spent sitting, viewing television, and using a computer during a day off might be unfavourably associated with ideal cardiovascular health [20]. Being sedentary is the main behavioural risk factor of all major non-communicable diseases, i.e. CVD, if appropriate action is not taken; deaths due to CVD are projected to rise further [21].

However, patients who stopped smoking also had the lowest LS. They included ‘disinclined actors’, patients who must adjust their lifestyles, 5 years after a coronary angiography, and adopt a behaviour that is perceived by them as unpleasant. The first hypothesis may be that the patients do not have a real ambition to stop smoking, ambivalence would exist between their intention and the behaviour, which they continued [22]. Additionally, patients whose attitudes were more aligned with social norms, such as stopping smoking, were more likely to accept the benefits of preventive behaviours [23]. The distance between the difficulties of making a modification and the ability to change behaviour would probably link with their low LS. Knowledge of the emotional impact of preventive behaviours such as physical exercise may be useful for policy interventions aimed at improving the health and daily life of patients [2]. The second hypothesis is in accord with recent research [24] which demonstrated that smoking reduction, sustained for at least 12 weeks, was not associated with a change in mental health, suggesting that reducing smoking was no better or worse for mental health than continuing smoking.

Among other interesting findings, the LS of INCCI patients (7.3/10; 69 years) was higher than in Luxembourgish patients (7.1/10; 65.5 years) living at home, two years after a stroke [25], and lower than the national LS indicator in Luxembourg (in 2013, 7.8), which was higher than that in the European Quality of Life Survey, 2012 (7.1 for EU-27) [7]. Relationships between socioeconomic conditions and LS are recognised. Our study revealed associations of gender, marital status and income with LS. Gender may include both social role and biological aspects. We observed that patients who are women and have a low to middle income had lower LS. The fact that the LS was lower in female than in male patients calls for further research on symptoms, and potential risk factors such as health-related behaviours, nutrition, leisure, etc. Our finding is not consistent with those of the European LS survey, which reported small gender difference in various countries. However, the study patients who lived in a couple were more satisfied, and slightly more satisfied than single people in various groups. We have no definitive explanation for these findings, but some relationships are well documented, for the whole population, in the recent report of the European Quality of Life Survey. People aged 25 or more who live with a partner have better LS than single people. This indicates that the emotional and social aspects of living in partnership are important to well-being [7]; in our sample, three out of four patients live in couples and their spouse or partner is probably the family caregiver.

An OECD working paper used the Gallup World Poll data to explore the determinants of well-being and examine the drivers of measures of affect (positive and negative states), as well as the determinants of LS that are more prevalent in the existing literature [26]. It reported that, overall, items relating to health status, personal security, and freedom to choose what to do with one’s life appear to have a larger impact on affect balance when compared to LS, while economic factors such as income and unemployment have a more limited impact [26]. Like patients living at home 2 years after stroke [25], we can postulate that the impact of socioeconomic factors would be greater for the study patients 5 years after their coronary angiography already facing disadvantages in lifestyle, and that the effect of health-related behaviours will be greater when they confront social disadvantages.

Cardiovascular disease affects health in combination with social, psychological and material factors [10, 13]. Contextualising our findings poses a challenge for a number of reasons, in particular the economic situation (as regards Luxembourg’s gross domestic product per inhabitant), and the fact that Luxembourg is one of the smallest European countries (502,500 inhabitants, area 2600 km^2^) and distances between the population and the health system are short. Care facilities are therefore geographically accessible to the whole population. The sociodemographic characteristics of the study patients (2/3 had an income of more than 36,000€) suggest that social and medical support focussed on community professional-oriented services is easily available. Location and income influence domiciliary care delivery: distribution of resources at local levels; financial constraints; and the application of eligibility criteria in providing medical and community services [11].

### This study has numerous strengths

The strengths reside in the population sample; the small size of the country made it possible to organize data collection at a national level. Our participation rate of 35.5 % is similar to 32.2 % in a previous study, also in Luxembourg [17]. This rate is a low estimate. Indeed, patients were contacted by mail and it was expected that relatives of deceased patients would inform us by return mail. We can assume that relatives of deceased patients were less motivated to respond than living patients, for this reason it is likely that the number of deceased patients is substantially higher and that the response rate is underestimated. Conducting a study 5 years after coronary angiography creates an opportunity to obtain valuable information. In this chronic phase, patients and their families may well have reorganised their daily lives and adapted to their new preventive behaviours. Such a study protocol remains rare because it is very expensive, and difficult to organise. Some patients died, now lived in institutions, had changed their residence (for example to live with a son or daughter), or failed to respond.

#### Some limitations concern the stronger finding for angina

For the authors of a recent study [3], one explanation involves a reporting bias. Angina is often established through self-reports of chest pain. Individuals with favourable views of their lives may be more likely to report favourable views of their health and have higher pain tolerance. All self-reported instances of angina were confirmed clinically in the present investigation, but the extent of its classification due to undiagnosed angina was probably dependent on self-report, which introduces a potential reporting bias according to one’s psychological outlook.

## Conclusions

Coaching patients with interventions that foster healthy attitudes can help sustain rehabilitation and improve LS. LS of women patients was lowest; this finding calls for further research on symptoms, and potential risk factors such as health-related behaviours. In addition, further research should examine whether therapeutic programs can reduce the distance between the ability to modify an intention and the ability to change behaviour. Health promotion programs would benefit from targeting factors that moderate the unfavorable intention-behaviour relationship. Our findings reflected the trends of the last report of Eurofound, which observed that the most important predictor of LS is health [7], but point to the existence of a social gradient on which the most socially and economically advantaged group have better health and well-being, and lower rates of illness and death than disadvantaged groups [27]. They also reflected the WHO recommendations on the adverse health consequences of risk factors as a basis for behaviour change or risk factor modification in individuals [9, 21].
